# Evaluation of 1D and 2D Deep Convolutional Neural Networks for Driving Event Recognition

**DOI:** 10.3390/s22114226

**Published:** 2022-06-01

**Authors:** Álvaro Teixeira Escottá, Wesley Beccaro, Miguel Arjona Ramírez

**Affiliations:** Department of Electronic Systems Engineering, Polytechnic School, University of São Paulo, São Paulo 05508-010, Brazil; alvaro.escotta@usp.br (Á.T.E.); miguel@lps.usp.br (M.A.R.)

**Keywords:** driving events analysis, time series analysis, recurrence plot, machine learning, artificial neural networks, convolutional neural networks, deep learning

## Abstract

Driving event detection and driver behavior recognition have been widely explored for many purposes, including detecting distractions, classifying driver actions, detecting kidnappings, pricing vehicle insurance, evaluating eco-driving, and managing shared and leased vehicles. Some systems can recognize the main driving events (e.g., accelerating, braking, and turning) by using in-vehicle devices, such as inertial measurement unit (IMU) sensors. In general, feature extraction is a commonly used technique to obtain robust and meaningful information from the sensor signals to guarantee the effectiveness of the subsequent classification algorithm. However, a general assessment of deep neural networks merits further investigation, particularly regarding end-to-end models based on Convolutional Neural Networks (CNNs), which combine two components, namely feature extraction and the classification parts. This paper primarily explores supervised deep-learning models based on 1D and 2D CNNs to classify driving events from the signals of linear acceleration and angular velocity obtained with the IMU sensors of a smartphone placed in the instrument panel of the vehicle. Aggressive and non-aggressive behaviors can be recognized by monitoring driving events, such as accelerating, braking, lane changing, and turning. The experimental results obtained are promising since the best classification model achieved accuracy values of up to 82.40%, and macro- and micro-average F1 scores, respectively, equal to 75.36% and 82.40%, thus, demonstrating high performance in the classification of driving events.

## 1. Introduction

The growth of vehicles in circulation has severe consequences, including increases in congestion, accidents, and environmental damage. The “Global status report on road safety” launched by the World Health Organization (WHO) in 2018 indicated that the number of annual road traffic deaths reached the mark of 1.35 million [1]. In addition, road-traffic injuries became the main cause of deaths of people aged 5–29 years, especially pedestrians, cyclists, and motorcyclists.

Among the risk factors associated with traffic accidents, the main ones deal directly with issues related to the behavior of drivers, affected by aggressiveness, distraction, drowsiness, and drunkenness. Potentially, aggressive behavior is one of the most recurrent causes that leads to unsafe traffic conditions and therefore fatalities [2]. Thus, monitoring a driver’s profile and, in particular, driving events has become a significant contribution in attempts to increase road safety.

The acquisition of steering behavior can be essentially performed by inertial motion sensors (i.e., accelerometers, gyroscopes, and magnetometers) connected to vehicles [3,4]. Some systems use data collected on vehicle communication buses, for example, Controller Area Network (CAN), Local Interconnect Network (LIN), and Flexray [5]. In these cases, it is possible to process other signals, such as the engine rotation, fuel consumption, oxygen level (measured by the lambda sensor), etc. In addition, it is also possible to apply computer vision techniques to obtain data related to the visual aspects of drivers [6,7] and roads. However, visual resources, based on computer vision techniques, have certain limitations, such as the violation of the user’s privacy, the restriction in the range of movement, and interference from the external environment.

Many papers studied the topic of driving behaviors [8]. Wang et al. [9] presented three deep-learning methods based on CNN, Long Short-Term Memory (LSTM), and Gated Recurrent Unit (GRU) to recognize aggressive driving behaviors. In addition, an evaluation of ensemble classifiers built with the proposed methods, was conducted. The signals used were acceleration, angular velocity, vehicle speed, distance and relative speed between the vehicle and rear objects. The accuracy values obtained by the CNN, LSTM, and GRU models were 74.33%, 73.67%, and 75.33%, respectively. The best performance of the ensemble classifier, based on LSTM, had an accuracy of 90.50%.

Moukafih et al. [10] proposed a Long Short-Term Memory-Fully Convolutional Network (LTSM-FCN) to evaluate aggressive behavior using combined features, including vehicle speed, acceleration, angular speed, car position relative to lane center, car angle relative to lane curvature, road width, distance to ahead vehicle, and time of impact to ahead vehicle. The best performance, an F1 score of 95.88%, in differentiating four classes of driving was achieved with a time window length of 5 min.

Ma et al. [8] evaluated four models based on Gaussian Mixture Model (GMM), Partial Least Squares Regression (PLSR), Wavelet transformation, and Support Vector Regression (SVR) to identify aggressive driving behavior. GMM attained the best results on average, based on the F1 score. Otherwise, PLSR achieved the best results when multi-source datasets (i.e., accelerometer and gyroscope datasets) were combined, obtaining an F1 score equal to 0.77.

Shahverdy et al. [2] classified driving styles, such as aggressive, distracted, drowsy, and drunk driving with 2D CNNs on images constructed from recurrence plots. Alkinani et al. [6] detected ten classes of distracted drivers with feature extraction based on deep CNN models and *k*-Nearest Neighbors (KNN) and Support Vector Machine (SVM) classifiers.

Another advancement lies in the increased capacity of imitation of driving styles, especially for autonomous vehicles. Zhou et al. [11] proposed a model based on generative adversarial imitation learning that can learn the strategy from drivers’ demonstrations. Hu et al. [12] described an end-to-end method for automated lane changing based on Light Detection And Ranging (LIDAR) information. Other references and a comprehensive review of vehicle driving behavior were described in [13]. Additionally, the authors presented a systematic review of the existing research on vehicle behavior prediction models.

As mentioned above, deep models have been applied in the recognition of driving style and behavior. Thus, evaluating the strategies for obtaining signals, pre-processing, and representation for input into deep models is essential to evaluate how this intelligence can be embedded in vehicles in the next few years. For this purpose, acceleration and angular velocity signals obtained by IMU (accelerometer and gyroscope sensors) are used in this work.

The processing of these signals is performed considering only the signal components that have the greatest discriminatory potential to categorize the events. The classification models are based on 1D and 2D CNN, implementing the AlexNet architecture with minor modifications, such as adapting the size of inputs and outputs. The signals from IMU sensors are transformed into an image-like representation of recurrent states using recurrence plots and then used as inputs of the 2D CNN model. The main contributions of the paper can be summarized as follows:

- A description of a setup using in-vehicle sensors and a methodology for obtaining data from driving events, including building a labeled dataset.
- The development of two CNN-based models for high accuracy classification of driving events. For each model, the performance analysis is conducted using the accuracy and F1 score metrics.
- We verify that the adapted version of the AlexNet model to its one-dimensional version (i.e., with 1D convolutional layers) achieves similar performance to the main models for multivariate time series classification.
- A comparative analysis of 1D and 2D models is conducted. In addition, the comparative performance is validated using statistical tests.

The remainder of this manuscript is structured as follows: Section 2 briefly presents the representation of time series using recurrence plots and the advantages of CNNs. The section also exemplifies some state-of-art deep-learning models and introduces the AlexNet architecture. Section 3 describes the method and infrastructure used to construct and preprocess the dataset. Section 4 discusses the results obtained, and Section 5 presents our conclusions.

## 2. Time Series Representations and Convolutional Neural Networks

### 2.1. Recurrence Plot

Recurrence analysis, introduced by Eckmann et al. [14], is a graphical method of representation designed to highlight hidden dynamical patterns, data structural changes, and nonstationarity. The recurrence plot obtained with the recurrence matrix r is the pairwise distance between the trajectories ${\vec{x}}_{i}$ and ${\vec{x}}_{j}$ calculated by

(1) $$R_{i,j}=\Theta \left(\varepsilon -∥{\vec{x}}_{i}-{\vec{x}}_{j}∥\right),\;i,j=1,\ldots ,N,$$

where Θ is the Heaviside function, ε is the threshold, *N* is the length of the time series, and $∥\cdot ∥$ is a defined norm (e.g., $L_{2}$, $L_{1}$ or $L_{\infty }$ norm). In other words, establishing a threshold, the darkened pixel (black dot) will only appear where the corresponding distance is below or equal to ε. Vectors compared with themselves compute to distances of zero, which can be seen by a line of identity in recurrence plots ($r_{i,i}{=1|}_{i=1}^{N}$).

Estimating a suitable value for the threshold is not a trivial task [15]. The literature presents several studies to define threshold values, where some are based on practical rules, for example, defining a percentage of the maximum distance [16]. Others include choosing a value based on the density of recurrence points [17] and/or a value five-times greater than the standard deviation of the observational noise [18].

Otherwise, it is also usual to construct a symmetric matrix of distances D by calculating the distances between all pairs of vectors, as

(2) $$d_{i,j}=∥{\vec{x}}_{i}-{\vec{x}}_{j}∥,\;i,j=1,\ldots ,N.$$

In this case, each distance value can be associated with a grayscale color, whose contrast corresponds to the magnitude of the values in a two-dimensional array. This strategy is sometimes called unthresholded recurrence plot and was used in this article to represent the time series obtained with the IMU sensors.

Other topologically equivalent representation by means of the method of time delays was proposed by Takens [19]. Thus, for a given univariate time series $\left(x_{1},\ldots ,x_{N}\right)$, the extracted trajectories, ${\vec{x}}_{i}$, are given by

(3) $${\vec{x}}_{i}=\left(x_{i},x_{i+\tau },\ldots ,x_{i+(M-1)\tau }\right),\;{\vec{x}}_{i}\in R^{M},$$

where *M* is the dimension of the trajectories and τ is the time delay. In this case, the recurrence plot is calculated in every point of the phase space trajectory of the embedded vectors ${\vec{x}}_{i}$ and ${\vec{x}}_{j}$—that is i,j=1,…,N−(M−1)τ.

Different approaches can be proposed for evaluating the patterns generated by the recurrence plots. Eckmann et al. [14] described small- and large-scale patterns (i.e., texture and typology patterns, respectively) that can be obtained with the recurrence plots [20]. The texture represents small-scale structures, such as diagonal lines, dots, vertical and horizontal lines. Otherwise, the typology characterized as periodic, drift, homogeneous, and disrupted represents the global structure.

### 2.2. Convolutional Neural Networks

In our investigation, we studied the learning of both time series and recurrence plots with CNNs for driving events recognition. CNNs are specifically designed to process data that has a known, grid-like topology [21]. These models have been widely used for applications such as image processing [22,23,24,25], video processing [26], denoising [27], speech processing [28,29,30,31], etc.

CNNs have three characteristics that help improve a machine-learning system [21,32]. First, CNNs typically work with sparse interactions, especially by making the kernel smaller than the input. Secondly, differently from traditional neural networks, CNNs use the same parameter for more than one function in the model. Thus, the filters (also known as kernels) can be applied at every position of the input, excluding possible boundaries depending on the parameter design. Finally, the parameter sharing property leads to the equivariance to translation, which is the ability to learn spatial patterns. This important aspect of CNNs allows the model to extract features from data series and images independently of temporal or spatial location.

There are groundbreaking CNN architectures that have been proposed to improve the accuracy and to reduce the computational cost. Some examples are: AlexNet, VGG-16 Net, Residual Network (ResNet) 18, ResNet 50, InceptionNet, InceptionNet V2/V3, and more recently, DenseNet and EfficientNet.

AlexNet [33] was the pioneer in deep CNN models and refocused the research on deep learning after winning the ILSVRC-2012 ImageNet competition. Although there have been several improvements in the deep CNN models since the introduction of AlexNet in 2012, the way that the architecture was carefully crafted, the use of Rectified Linear Unit (ReLU) activation functions, and the strategy used to avoid overfitting by regularization show that this model is still an excellent choice. In addition, as indicated in recent studies, AlexNet model and AlexNet-inspired architectures are widely applied in pattern recognition tasks [34,35,36,37,38].

## 3. Materials and Methods

### 3.1. Data Acquisition

The driving event data were collected under real conditions. In the experiments, the signals from each event were obtained by sampling the inertial motion sensors of a smartphone using the *phyphox* app, developed by RWTH Aachen University [39]. The *phyphox* is an open source, free, and ad-free application for experiments with smartphone sensors and devices, including accelerometers, gyroscopes, magnetometers, microphones, loudspeakers, global positioning systems (GPS), and others. It is possible to collect simultaneous data from different sensors allowing data logging and real-time observation both locally and remotely.

**Figure 1:**
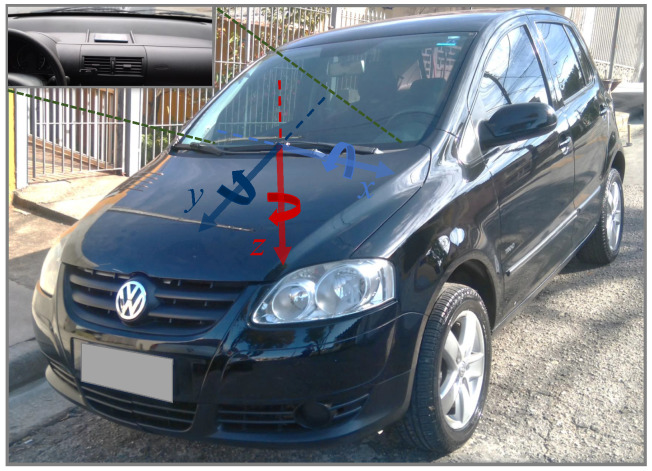
Smartphone installation location (in detail) and coordinate reference system *x*, *y*, and *z*. The *x* axis points to left and is perpendicular to the vehicle plane of symmetry. The *y* axis points forward and is parallel to the vehicle plane of symmetry. The *z* axis extends downwards. The pitch, roll, and yaw are the counterclockwise (right-handed) rotations about the *x*, *y*, and *z* axes, respectively.

The driving events experimentally performed were labeled as non-aggressive events, aggressive right and left turns, aggressive right and left lane changes, aggressive braking, and aggressive accelerations. The experiments were conducted by a single and experienced driver in three trips of approximately 25, 16, and 27 min. The driving events were performed using a single vehicle, 2010 Volkswagen Fox 1.0, under conditions of partly cloudy weather, dry track, and regular asphalt. The data were sampled with a smartphone model Xiaomi Redmi Note 8 Pro with Android version 10 [40]. The smartphone was affixed to the instrument panel of the vehicle in landscape orientation, maintained no movement or operation during the trips. Figure 1 shows the coordinate system used to reference the sensors. We extended the number of drivers by involving an additional dataset [41] composed of four trips (experimented with a 2011 Honda Civic) performed by two drivers executing the same lateral (right and left turn as well as right and left lane change) and longitudinal (braking and acceleration) driving events.

The calibration of the IMU sensors was previously verified with *phyphox*. As many recent Android devices do not provide a native calibration utility, the IMU sensors can be calibrated with free third-party apps if necessary.

There are many definitions of aggressive driving behavior. However, it is possible to associate this behavior with some specific actions, such as excessive speed, repeated and frequent lane changes, inconsistent and excessive acceleration, and sudden braking. To identify that the driver’s behavior is aggressive—that is, that it differs from a normal driving pattern, it is necessary to define a reference driving pattern that can be called safe, normal, or even non-aggressive [42]. Thus, the experiment was based on events that emulate the attributes defined for aggressive and non-aggressive driving, based on the driver’s experience.

Data from the inertial motion sensors (i.e., accelerometer and gyroscope) were collected with a sampling frequency of 400 Hz. In order to construct a dataset that represents real-world driving events (e.g., acceleration, breaking, turning, and lane changes), we obtained 169 events, subdivided into 26 non-aggressive events, 25 aggressive right-turn events, 23 aggressive left-turn events, 29 aggressive lane change events to the right, 23 aggressive lane change events to the left, 22 aggressive braking events, and 21 aggressive acceleration events.

The non-aggressive events replicate the events of accelerating, braking, lane changing, and turning; however, with less intensity and greater caution during movements. These events were labeled by the driver, recording the start and end of the events with an audio recorder app running in the background on Android throughout the trip period. With this arrangement, someone listening to the recorded audio later could exactly mark what driving event was conducted and when the event started and ended.

According to the literature, some studies assigned scores to aggressive behaviors (e.g., from 1 to 5 [43], or from 1 to 10 [44], representing the least aggressive to most aggressive behaviors). Here, the data labeling was assigned using binary scores, namely aggressive and non-aggressive. Although some events appear to be a combination of two steering actions (e.g., deceleration while turning or acceleration and lane changing), these events will be classified according to the class they most resemble; however, most importantly, they will be labeled as aggressive events or not.

As previously indicated, we relied on the driver’s experience and perception on how to perform an aggressive or non-aggressive maneuver in order to label the events. We also aimed to collect a balanced number of events for each class and made our best effort to evenly ensure that events were repeatable. We discarded labeling some events due to errors during the experiments, such as events that were started but not completed for security reasons. The additional dataset incorporated 69 more labeled events.

The data for each driving event present triaxial information of the linear acceleration (acceleration without the influence of gravity) measured in m/s^2^, and the angular velocity, measured in rad/s. In general, aggressive behavior was emulated by driving events with severe acceleration and deceleration characteristics as well as abrupt changes in direction (wide turns). As above-mentioned, non-aggressive events were represented by different driving events under the conditions of acceleration, deceleration, and tight/smooth turns.

### 3.2. Data Preprocessing

IMU sensors are affected by high-frequency noise that can be attenuated by using a filtering strategy [45]. Hence, after segmented and labeled, the data were preprocessed using the Savitzky–Golay smoothing filter. This filter fits a sequence of samples to a polynomial in the least-squares sense [46]. Two parameters are used to perform the adjustment, the window size that sets the number of data points (a positive odd integer) and the degree of the polynomial (less than window size −1).

The best fit results were obtained with frames of 21 samples and third degree polynomial interpolation. Both parameters were empirically estimated. The order of the polynomial fitting was kept lower to introduce the least possible distortion into the original signal and to preserve the meaningful information contained in the original one, however, with less noise and/or fluctuations.

### 3.3. Recurrence Plot Representation

As described in Section 2, the recurrence plots were constructed by computing distances between all pairs of embedded vectors to obtain two-dimensional representations of the time series of acceleration in the *x* and *y* directions and angular velocity in the *z* direction. The recurrence plot can be visualized as an image formed by a square matrix, whose entries correspond to the recurrence estimates.

The implementation of the recurrence plots was conducted with the Python library *pyts*, which includes resources for transforming time series into 2D representations. This library enables the construction of time series recurrence plots by applying the time delay incorporation technique. The parameters necessary to obtain the recurrence plots are the time delay (i.e., the time gap between two back-to-back points of the trajectory) and the dimension of the trajectory, *M*. In our proposal, we assign a unitary value for the embedding dimension and time delay as originally proposed for recurrence plot representation. In addition, the library uses the Euclidean distance ($L_{2}$ norm).

### 3.4. Model Implementation and Hardware

The recognition of driving events is performed using one-dimensional (1D-CNNs) and two-dimensional (2D-CNNs) convolutional neural networks. For this, the proposed models were based on the one-dimensional (1D-AlexNet) and two-dimensional (2D-AlexNet) AlexNet architecture [33]. The 1D-AlexNet model demonstrated good performance for time-series data classification [47,48], and the 2D-AlexNet model is a renowned architecture for image processing, mainly due to its robustness.

The model consists of eight layers with adjustable parameters. The first five ones are convolutional layers and the last three ones are Fully Connected (FC) layers. After the first, second, and fifth convolutional layers, there are max-pooling operation layers. As mentioned above, the architecture is also composed of three FC layers and the network output is normalized by the softmax function. The activation function used in the convolutional and FC layers is the ReLU. In addition, dropout layers are placed before and after the first FC layer to avoid overfitting.

Based on the AlexNet architecture, the first convolutional layer filters the input data with 96 kernels of size 11×11 and stride of 2×2. The second convolutional layer receives as input the reduced (i.e., downsampled) data from the first convolutional layer and applies 256 kernels of size 5×5. The third, fourth, and fifth convolutional layers are connected without any normalization layer or pooling operation. The third and fourth convolutional layers have 384 kernels of size 3×3, and the fifth convolutional layer has 256 kernels of size 3×3. The model also has two dropout layers with regularization of p=0.5 to prevent overfitting. The first two FC layers have 4096 neurons each. Finally, the output layer has seven dimensions, which is the number of labeled driving events. The loss function used is the cross entropy.

**Figure 2:**
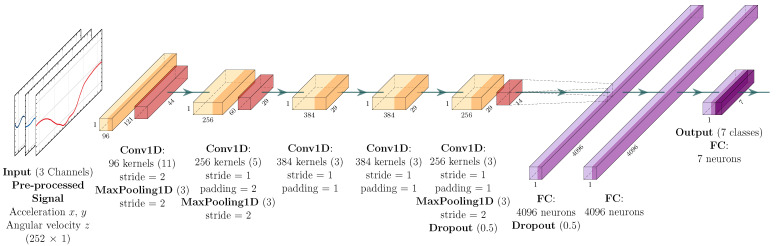
1D-AlexNet architecture. Each block describes the parameters assigned to the respective layers, as for blocks “Conv1D” and “MaxPooling1D”, where the first value corresponds to the size of the filter.

**Figure 3:**
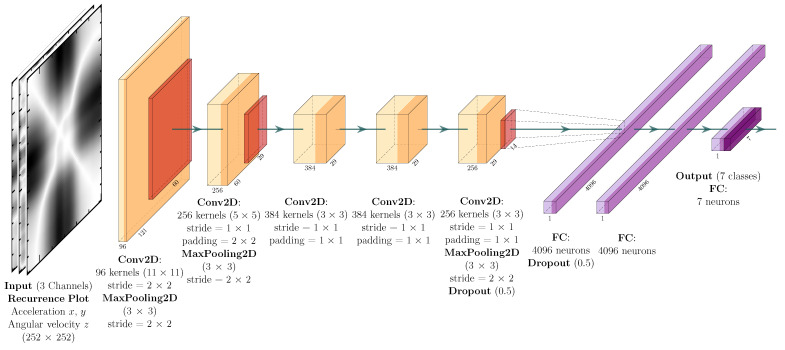
2D-AlexNet architecture. Each block describes the parameters assigned to the respective layers, as for blocks “Conv2D” and “MaxPooling2D”, where the first value corresponds to the size of the filter.

The 1D-AlexNet architecture has the same sequential design and the same parameters previously described. However, the 2D convolutions are substituted by 1D convolution operations as well as the other network parameters. Figure 2 and Figure 3 present the 1D-AlexNet and 2D-AlexNet network architectures, respectively, with the description of the layers’ parameters and dimensions.

Some events present approximately 4032 samples per window (10.08 s duration considering the sampling rate of 400 Hz). As we are dealing with events with low frequency, the data were downsampled by 16 after applying an eighth-order Chebyshev type I low-pass filter, reducing the number of samples per driving event and thus reducing the training time of the network without losing significant information.

The input data of the 1D-AlexNet model are composed of three channels corresponding to the proposed time series (linear acceleration in *x* and *y* and angular velocity in *z*), each one with 252 samples. Likewise, the input data of the 2D-AlexNet model is composed of three channels, each channel being related to the recurrence plots of the proposed signals. Each recurrence plot corresponds to a 252×252 pixel image. In both cases, signals shorter than 10.08 s (i.e., 252 samples considering the sampling rate of the downsampled signal equal to 25 Hz) were filled with the zero-padding technique.

Training and performance evaluation were performed by applying the *k*-fold cross-validation strategy. The proposed models were trained and validated with *k* equal to 5 and 10. Leave-one-out cross-validation (LOOCV) was also performed. The models are implemented using the PyTorch machine learning library and trained for 1000 epochs with Adam optimization algorithm and learning rate of 0.001. The training was processed with full batch and lasts approximately 2 min and 17 s for the 1D-AlexNet model and 1 h and 38 min for the 2D-AlexNet model on a Google Colab Pro virtual machine that provides 27.3 GB of RAM, an Intel Xeon CPU 2.20 GHz processor with four cores, and a Tesla P100-PCIE GPU accelerator with 16 GB.

### 3.5. Evaluation Metrics and Training Schemes

To evaluate the trained models, we applied the accuracy and F1 score metrics. The accuracy was calculated as

(4) $$\mathrm{Accuracy}=\frac{TP+TN}{TP+TN+FP+FN\prime }$$

where TP, TN, FP, and FN are, respectively, the True Positive, True Negative, False Positive, and False Negative elements. The accuracy is directly computed from the confusion matrix and represents how much the model is correctly predicting on the entire set of data. In general, accuracy is not indicated for imbalanced classification; however, as we indicated in Section 3.1, we constructed a balanced database.

The F1 score also assesses the performance of classification models by the harmonic mean of

(5) $$\mathrm{Precision}=\frac{TP}{TP+FP}$$

and

(6) $$\mathrm{Recall}=\frac{TP}{TP+FN\prime }$$

calculated as

(7) $$F1\;\mathrm{Score}=2\frac{\mathrm{Precision}\times \mathrm{Recall}}{\mathrm{Precision}+\mathrm{Recall}}.$$

In a multi-class setting, one can calculate the F1 score for each class in a One-vs-Rest (OvR) approach, determining the metrics for each class separately. To average the multiple per-class metrics in order to describe the overall performance, one can apply the methods of micro-, macro-, and weighted-average. In the micro-average method, one sums up the individual TP, FP, and FN of the model for different sets and applies them to obtain the metrics. The macro-average method takes the average of the precision and recall of the model on different sets. Finally, the weighted-average takes the average of the precision and recall of the model on different sets weighted by support (i.e., the number of true instances for each label). In some cases, the weighted-average can result in an F1 score that is not between the precision and recall.

The low number of driving event samples in the dataset considerably reduces the possibility of partitioning the data into training and test/validation groups used during model learning. In this condition, the results would depend on random choices of the data, and the generalizability of the models could be inadequate. The models could be sensitive to the data used, and a small change in the training dataset could result in a significant difference in the obtained models. The evaluation of the models becomes more robust using the *k*-fold cross-validation that allows more variations of the possible divisions of the data in training and test/validation, although it is a method that consumes more time than the hold-out.

## 4. Results and Discussion

### 4.1. Driving Events

We verified that the linear acceleration data in the directions *x* and *y* presented a greater discriminatory potential for the categorization of the driving events since these events cause significant changes along these axes. Furthermore, to infer the lateral dynamics of the vehicle, as well as to differentiate the direction of occurrence of the events, the angular velocity data in the *z* direction can be used. Although there is a certain behavior of variation of the acceleration and angular velocity signals in all other directions, these signals have less capacity to describe the events that have occurred. Therefore, instead of using the triaxial data of each sensor, only two axes of the accelerometer and one of the gyroscope were considered.

**Figure 4:**
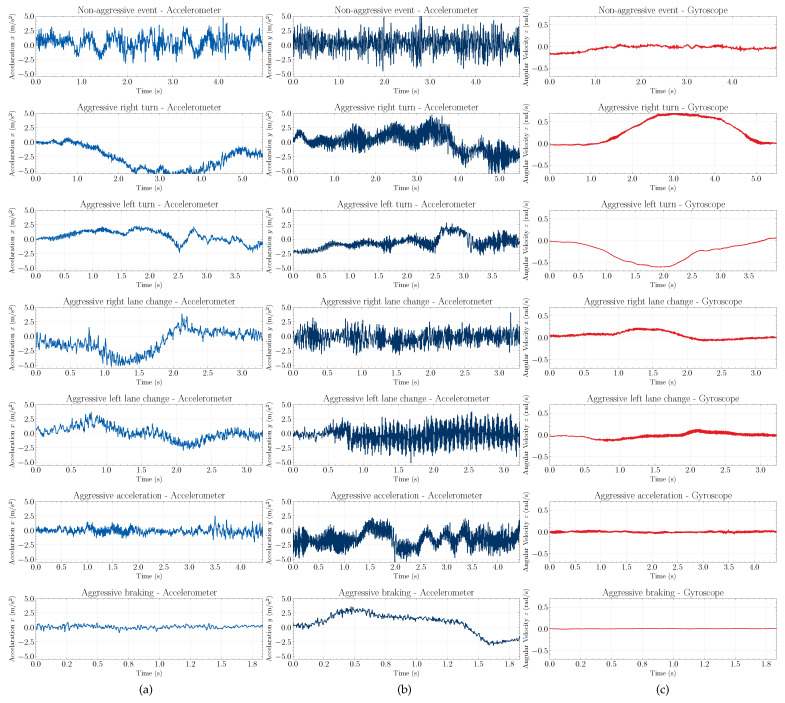
Raw data of linear acceleration in the *x*, first column (**a**), and *y*, second column (**b**), direction and angular velocity in *z*, third column (**c**), for non-aggressive events (first row), aggressive right and left turn (second and third rows), aggressive left and right lane change (fourth and fifth rows), aggressive braking (sixth row), and aggressive acceleration (seventh row), respectively.

**Figure 5:**
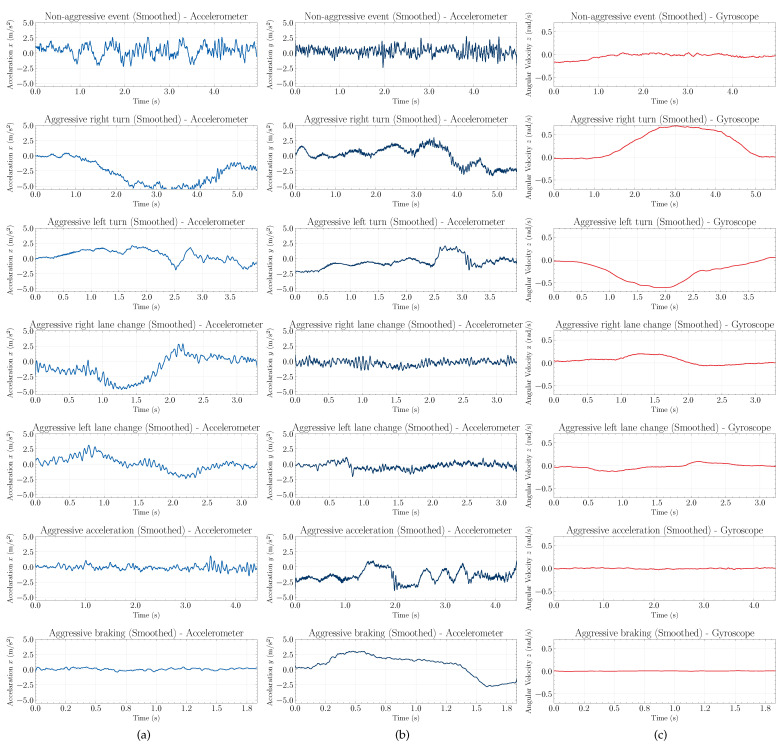
Smoothed data of linear acceleration in the *x* direction, first column (**a**), *y* direction, second column (**b**), and angular velocity in the *z* direction, third column (**c**), for non-aggressive events (first row), aggressive right and left turn (second and third rows), aggressive left and right lane changing (fourth and fifth rows), aggressive braking (sixth row), and aggressive acceleration (seventh row), respectively.

Figure 4 shows one set of raw collected data of linear acceleration in the *x* and *y* directions and angular velocity in the *z* direction. Figure 5 shows the same events now smoothed by the Savitzky–Golay filter. It is noted that, after preprocessing the data, even though there is a decrease in terms of the signal amplitude, we verified that the behavior of the events is maintained. It improves the visualization of the patterns and trends of the signals. In this way, it is possible to identify different characteristics among the signals of the presented events.

Based on the acceleration signals in the *x* and *y* directions, we observed that the non-aggressive event was characterized by low-amplitude oscillations along the event window. This is because non-aggressive events present smooth accelerations, decelerations, and steering.

Otherwise, aggressive braking and aggressive acceleration events present more significant variations in the *y* direction of the acceleration signal, respectively, forward and backward. When the brakes are pressed, it is expected that anything not rigidly fixed to (or in) the vehicle will tend to continue moving forward (i.e., anything in motion tends to stay in motion unless acted on by an outside force), thereby, indicating positive values of the acceleration in the *y* direction. Otherwise, when the car starts moving forward, anything not rigidly fixed experiences a backward acceleration, which can be seen by negative values of acceleration in the *y* direction.

We observed that aggressive turns and aggressive lane changes events present more significant variations in the *x* axis of the linear acceleration signal, as this is the direction substantially affected by these movements. The aggressive aspect was observed with the increase of amplitude in the signal since non-aggressive movements have almost the same shape with less variation in amplitude.

Based on the angular velocity signals in the *z* direction, we observed that the non-aggressive events, aggressive braking, and aggressive acceleration do not present a marked variation, since these events do not change direction. In these events, the signals are characterized by small oscillations. On the other hand, events, such as turns and lane changes present distinctive behaviors, since the angular velocity data capture the change in the orientation of the movement during the execution of the driving event.

In general, the typical curve of aggressive acceleration presents at first a higher slope, followed by levels of constant and decreasing acceleration that represent the gear changes. Otherwise, aggressive braking initially has a higher slope deceleration followed by a curve that returns to an initial value, usually zero.

The lane change and turn events also have specific patterns on the *z* axis of the gyroscope. Assuming that the vehicle is making a right lane change, the signal in the *z* axis of the gyroscope increases to a high value and then decreases back to a lower value—that is, the steering wheel is turned twice, once to the right and once to the left to correct the vehicle’s direction. The pattern is reversed for the left lane. In the turn events, the steering wheel is turned to one side only; thus, for the right turn event, there is an increase in the value of the gyroscope followed by a return to zero. The pattern is reversed for the left turn event.

**Figure 6:**
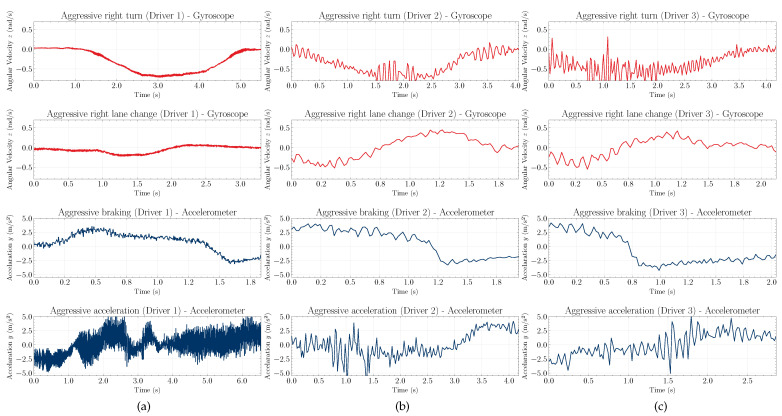
Examples of driving events performed by three different drivers: (**a**) first, (**b**) second, and (**c**) third driver. The angular velocity in *z* (first and second rows) for aggressive right turn and aggressive right lane change, and linear acceleration data in the *y* (third and fourth rows) for aggressive braking and aggressive acceleration.

Figure 6 depicts examples of driving events performed by three different drivers. The most significant axes were chosen to represent aggressive right turn, aggressive right lane change, aggressive braking, and aggressive acceleration events. As already mentioned, the events follow a time-series pattern, which can vary, in general, in terms of amplitude (depending on the greater or lesser aggressiveness of the driver) or in the duration of the event. However, the waveforms presented in Figure 6 suggest that the behavior of events follows a defined pattern for different drivers with different vehicles.

### 4.2. Analysis of Recurrence Plots

**Figure 7:**
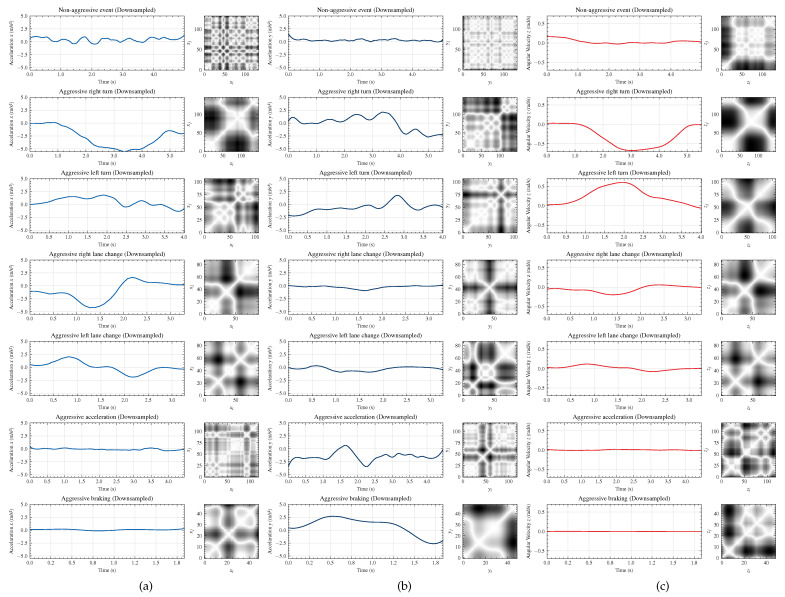
Time series and recurrence plots (on the right side of each driving event) of linear acceleration signals in the *x* and *y* directions, first (**a**) and second (**b**) columns, and angular velocity in the *z* direction, third column (**c**).

**Figure 8:**
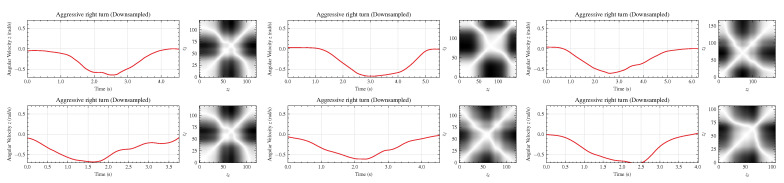
Time series and recurrence plots (on the right side of each driving event) of the angular velocity signal in the *z* direction for six different aggressive right turn events.

Figure 7 shows the recurrence plots corresponding to the preprocessed signals of linear acceleration in the directions *x* and *y*, and angular velocity in the *z* axis. The patterns of the time series and recurrence plots for driving events of the same class are repetitive. For example, Figure 8 depicts the time series and recurrence plots corresponding to the angular velocity signal in the *z* direction for six different data samples of aggressive right turn events. One can verify the repetitive aspect of the recurrence plots. The same can be seen for the acceleration signals. In general, repetitive patterns occur for all classes, demonstrating that the recurrence plots are good representations of the signals.

Some patterns can be observed in the recurrence plots of Figure 7. One of the typical aspects is related to the abrupt changes in the dynamics, which produce white bands in the recurrence plots and are typical characteristics of aggressive event actions. In addition, the visual aspects of the recurrence plots can be described using characteristic patterns, such as typologies. For aggressive events that present greater variation in the amplitude of the acceleration and angular velocity, the structure of the recurrence plot presents visual aspects of disrupted typology.

These characteristics are most evident for the acceleration signals in *x* and *y* and angular velocity in *z* for aggressive turn and aggressive lane change events, in addition to the acceleration signal in *y* for aggressive braking and aggressive acceleration events. On the other hand, non-aggressive event signals and events that present low dynamic range present a recurrence plot with a typical structure of homogeneous and drift typologies. These characteristics are observed, with more emphasis, in the signals of linear acceleration in *x* and angular velocity in *z* of the aggressive braking and aggressive acceleration events.

### 4.3. Analysis of Recognition Results of 1D-CNNs

The model 1D-AlexNet performance was evaluated by calculating the accuracy, macro- and micro-average F1 scores, and the results are presented in Table 1. Two different values of *k* were evaluated in order to analyze the sensitivity of this parameter in relation to the model evaluation scores. The accuracy estimated from the LOOCV for the 1D-AlexNet was 82.40% and micro- and macro-F1 equal to 82.40% and 75.36%, respectively. These results are close to the average of the cross-validated scores obtained with five-fold cross validation.

**Figure 9:**
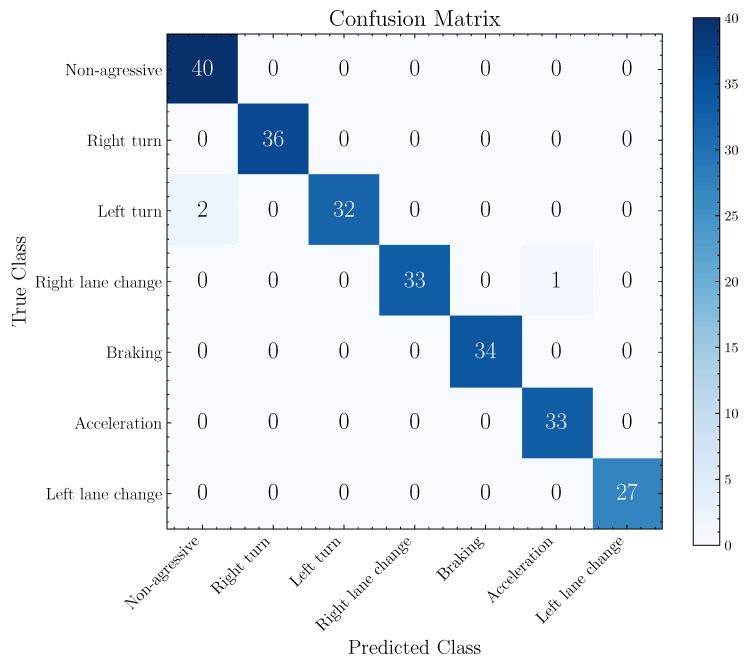
Confusion matrix of the 1D-AlexNet model (best model trained and validated with five-fold cross-validation). Confusion matrix obtained from the evaluation of the entire dataset.

The best general performance presents an accuracy of 93.61% referring to the evaluation of the 1D-AlexNet structure using the cross-validation with *k* equal to 5. Based on the model with the best result, Figure 9 presents its confusion matrix obtained by evaluating the entire database, visualizing the relation between predicted classes and real classes. The total number of parameters of 1D-AlexNet is 24,265,159, the same number of trainable parameters.

From the confusion matrix, illustrated in Figure 9, non-aggressive, aggressive right turn, aggressive braking, aggressive acceleration and aggressive left lane change events present a true positive rate (TPR) of 100%. Otherwise, the aggressive left turn and aggressive right lane change events present a TPR of 94.13% and 97.06%, respectively. The model presented incorrect predictions in two aggressive left turn events as non-aggressive events and one aggressive right lane change event as aggressive acceleration.

### 4.4. Analysis of Recognition Results of 2D-CNNs

The performance of the 2D-AlexNet model was also evaluated by calculating accuracy, micro- and macro-average F1 scores, and the results are summarized in Table 2. The accuracy estimated from the LOOCV for the 2D-AlexNet was 65.71% and micro- and macro-F1 equal to 65.71% and 56.04%, respectively. These results are close to the average of the cross-validated scores obtained with 10-fold cross validation.

**Figure 10:**
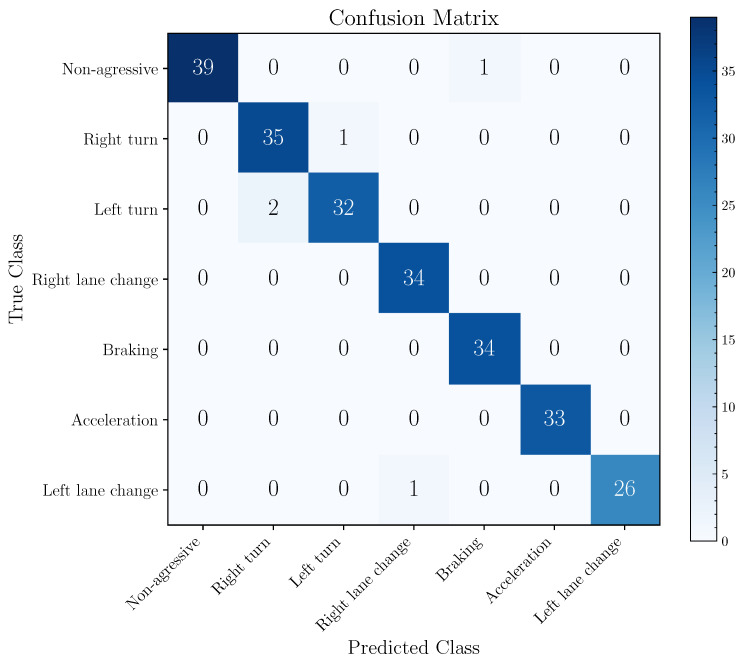
Confusion matrix of the 2D-AlexNet model (best model trained and validated with 10-fold cross-validation). Confusion matrix obtained from the evaluation of the entire dataset.

The best general performance presents an accuracy of 78.26% referring to the evaluation of the 2D-AlexNet structure using the cross-validation with *k* equal to 10. Based on this model, Figure 10 exhibits its confusion matrix calculated over the entire database. The total number of parameters and trainable parameters of 2D-AlexNet is equal to 58,310,023.

Figure 10 provides the description of the prediction errors. Aggressive right lane change, aggressive braking and aggressive acceleration events have a TPR of 100%. On the other hand, non-aggressive events, aggressive right and left turn and aggressive left lane change events present errors in the predictions, implying a TPR of 97.50%, 97.22%, 94.12% and 96.30%, respectively. The model presented five incorrect predictions, whose biggest errors occurred in the recognition of aggressive left turns as aggressive right turns.

The errors obtained by the 2D-AlexNet model are mainly associated with similar events, such as the recognition between aggressive turns and/or aggressive lane changes.

### 4.5. Statistical Significance Test

The results indicate that the 1D-AlexNet model overcomes the performance of the 2D-AlexNet model. To assess whether this result is consistent, we apply the hypothesis test to verify if the observed results obtained with 1D-AlexNet and 2D-AlexNet are statistically significant. The 5×2 cross-validation paired *t*-test (also known as Dietterich’s 5 × 2cv paired *t*-test) procedure was applied to compare the performance of the two classifiers [49]. We established as the null hypothesis that there was no significant difference between the result of 1D-AlexNet and 2D-AlexNet. As the alternative hypothesis, we set that there is a significant difference between the results of the classifiers. We tested the accuracy of the models as the parameter of comparison.

We employed data augmentation on signals of the original dataset with different methods, such as jittering (σ=0.03), scaling (σ=0.8), magnitude warping (with four knots at random magnitudes and σ=0.2), time warping (with four knots and σ=0.2), and window warping (window ratio equal to 0.1 and warping randomly selected from 0.5 or 2 times) [50]. As a final step, we performed the hypothesis test with 914 samples (238 from the original dataset and 676 slightly modified copies).

**Figure 11:**
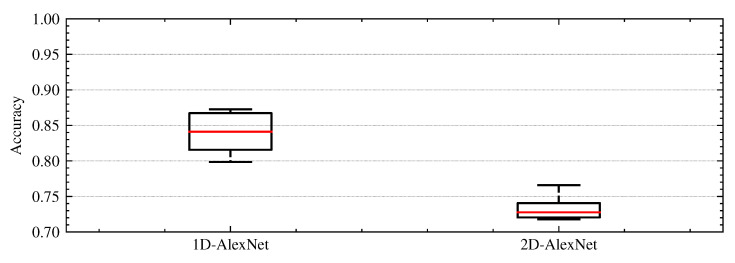
Box and whisker plot for the distributions of accuracy scores of 1D-AlexNet and 2D-AlexNet obtained with the Dietterich’s 5 × 2cv paired *t*-test (considering the augmented dataset). Boxes extending from the first quartile below to the third quartile above. The median line is in red, and whiskers extend to the minimum and maximum values.

Using the 5 × 2cv paired *t*-test, the *p*-value computed is equal to 0.0012 and when compared with the previously chosen significance level, in our case α=0.05 (5% significance level), is smaller than α. Thus, we rejected the null hypothesis and accept that there is a significant difference in the two models. Figure 11 shows a box and whisker plot that summarizes the distribution of accuracy scores. The results highlight that the 1D-AlexNet model is superior to its 2D version for the proposed task considering the conditions previously described.

### 4.6. Models Comparison and Transfer Learning

The AlexNet models were also compared with other six well-established models, namely Temporal Convolutional Network (TCN) [51], LSTM-FCN [52,53], Transformer [54,55], Time Series Transformer (TST) [56,57], automated mobile neural architecture search (MnasNet) [58], and EfficientNet-B4 [59].

TCN implements dilated casual 1D convolutions suitable for modeling sequential data. The dilation factor ensures large and flexible receptive field convolutions. Recently, TCN models outperformed Recurrent Neural Network (RNN)-based approaches in applications, such as recognizing human actions [60]. In its turn, LSTM-FCN enhanced the performance of FCN on the task of classifying univariate [52] or multivariate [61] time series sequences.

The Transformer implements an encoder–decoder structure architecture without recurrent connections. In short, the encoder uses a self-attention layer to obtain a representation of the input sequence and the decoder generates the output sequence one token at a time with an attention layer that helps focus on relevant parts of the input sequence [54].With a transformer-based framework, TST proposed an unsupervised representation learning of multivariate time series suitable for classification tasks [57].

MnasNet is a neural network based on CNN optimized for mobile devices with a good trade-off between accuracy and latency [58]. Finally, EfficientNet is state-of-the-art neural network architecture that presents a method to scale CNNs to achieve better accuracy and efficiency in terms of model parameters and floating-point operations per second (FLOPS) [59].

We tested the models with the same 914 samples previously described, splitting the dataset with the same procedure of 5 × 2cv. In addition, we evaluate the process of fine-tuning models pre-trained with the ImageNet dataset, especially the 2D-AlexNet, MnasNet, and EfficientNet-B4. In these models, the first and last layers were adapted to match the size input data (i.e., 252×252) and the number of classes (i.e., seven classes), respectively.

**Figure 12:**
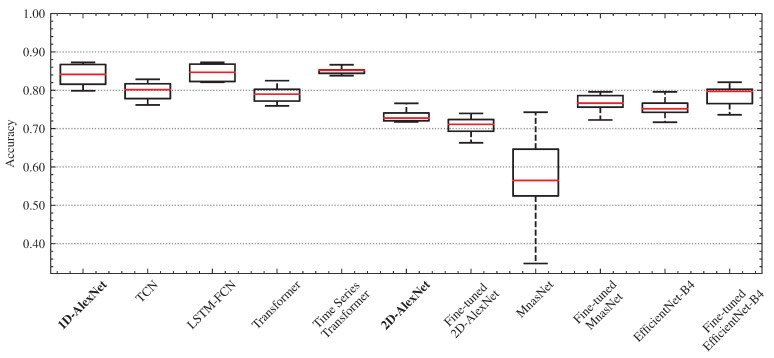
Box and whisker plot for the distributions of accuracy scores (considering the augmented dataset). Boxes extending from the first quartile below to the third quartile above. The median line is in red, and whiskers extend to the minimum and maximum values.

Figure 12 shows a box and whisker plot that summarizes the accuracy scores of all the models under consideration. The results indicate that the 1D models (1D-AlexNet, TCN, LSTM-FCN, Transformer, and TST) are, in general, more accurate when compared to 2D models (2D-AlexNet, MnasNet, and EfficientNet-B4). However, the fine-tuned pre-trained model EfficientNet-B4 demonstrated similar results to 1D model approach.

The MnasNet and the pre-trained 2D-AlexNet models presented the worst performances. In this case, the transfer learning strategy for 2D-AlexNet model is not as efficient when compared to the MnasNet and EfficientNet-B4 models. TST model achieved accuracy greater than 83.80% in all the iterations, highlighting its effectiveness in multivariate time series classification.

The 1D-AlexNet presented equivalent results compared to those obtained with the LSTM-FCN and TST models. When compared using 5 × 2cv paired *t*-test, one can see that there are no significant differences between the paired models since the *p*-values (p=0.9252 for the comparison between 1D-AlexNet and LSTM-FCN) and (p=0.2136 for the comparison between 1D-AlexNet and TST) are greater than α=0.05. One of the reasons for this is that the data is properly segmented and labeled, which improves the performance of the 1D-AlexNet during the training and validation. Future work will focus on the analysis of events that are partially or badly segmented.

## 5. Conclusions

Monitoring driving events has been explored due to the growing interest in systems that allow monitoring movement patterns. This paper presents two supervised deep-learning models based on 1D-CNNs and 2D-CNNs to classify aggressive and non-aggressive driving events. The best results of the 1D-AlexNet and 2D-AlexNet models reached accuracy values of up to 82.40% and 78.26%, respectively. Using *k*-fold cross-validation, the average accuracy for 1D-AlexNet and 2D-AlexNet was 81.97% and 64.35%, respectively. In addition, IMU sensors appeared as an attractive means to obtain in-vehicle data able to characterize driver behavior.

Based on the models and conditions presented throughout the article, the results indicate an advantage of the 1D-AlexNet over 2D-AlexNet, as confirmed by the statistical significance test. Furthermore, the 1D-CNN model had a lower computational cost, both for training and inference, a reduced number of parameters, and a direct application over the signals without the need to represent them using recurrence plots. Thus, despite CNNs being generally indicated to extract data from images, the one-dimensional approach presented better results both in terms of the classification and execution time for the proposed task.

We also compared the AlexNet models with other state-of-art architectures. The adapted version of the AlexNet model to its one-dimensional version achieved similar performance to LSTM-FCN and Time Series Transformer models under the conditions and restrictions of the proposed application.

The results are consistent for the evaluation of data collected through inertial motion sensors—in our case, accelerometers and gyroscopes. The data encompass the most recurrent driving events during vehicular conduct and characterize the aggressive behavior of drivers. It is noteworthy that the experiments conducted for data collection showed regular road conditions and a stable climate. Different road conditions (e.g., potholes and slopes) and weather conditions (e.g., rain and fog) are likely to impact driving behaviors.

The presented driving events recognition can be exploited in several applications, such as car insurance operators, car rentals, and shared services companies. There is also great potential for applications in intelligent transport systems, autonomous vehicles, the monitoring of road and vehicle conditions, and eco-driving.

## Figures and Tables

**Table 1:** Performance measures of the 1D-AlexNet model: accuracy, micro- and macro-average F1 scores. Average cross-validated scores across all subsections of the data.

Model	Cross-Validation	Accuracy (%)	Micro-F1 Score (%)	Macro-F1 Score (%)
1D-AlexNet	5-fold ^1^	81.97±7.59	81.97±7.59	80.75±8.13
	10-fold ^1^	78.55±10.54	78.55±10.54	72.69±13.02
	Leave-one-out ^2^	82.40	82.40	75.36

^1^ Classification results (mean ± standard deviation). ^2^ Classification results (mean).

**Table 2:** Performance measures of the 2D-AlexNet model: accuracy, micro-, and macro-average F1 scores. Average cross-validated scores across all subsections of the data.

Model	Cross-Validation	Accuracy (%)	Micro-F1 Score (%)	Macro-F1 Score (%)
2D-AlexNet	5-fold ^1^	63.45±6.84	63.45±6.84	60.56±6.71
	10-fold ^1^	64.35±10.28	64.35±10.28	57.27±11.95
	Leave-one-out ^2^	65.71	65.71	56.04

^1^ Classification results (mean ± standard deviation). ^2^ Classification results (mean).

## Data Availability

The data presented in this study are available on request from the corresponding author.
